# Neuroimaging and machine learning in eating disorders: a systematic review

**DOI:** 10.1007/s40519-025-01757-w

**Published:** 2025-06-01

**Authors:** Francesco Monaco, Annarita Vignapiano, Benedetta Di Gruttola, Stefania Landi, Ernesta Panarello, Raffaele Malvone, Stefania Palermo, Alessandra Marenna, Enrico Collantoni, Giovanna Celia, Valeria Di Stefano, Paolo Meneguzzo, Martina D’Angelo, Giulio Corrivetti, Luca Steardo

**Affiliations:** 1 Department of Mental Health, Azienda Sanitaria Locale Salerno, Salerno, Italy; 2 European Biomedical Research Institute of Salerno (EBRIS), Salerno, Italy; 3 University of Padova, Padua, Italy; 4 University Telematica Pegaso, Naples, Italy; 5 University “Magna Graecia” of Catanzaro, Catanzaro, Italy

**Keywords:** Neuroimaging, Machine learning, Eating disorders, Biomarkers, Predictive analytic

## Abstract

**Purpose:**

Eating disorders (EDs), including anorexia nervosa (AN), bulimia nervosa (BN), and binge eating disorder (BED), are complex psychiatric conditions with high morbidity and mortality. Neuroimaging and machine learning (ML) represent promising approaches to improve diagnosis, understand pathophysiological mechanisms, and predict treatment response. This systematic review aimed to evaluate the application of ML techniques to neuroimaging data in EDs.

**Methods:**

Following PRISMA guidelines (PROSPERO registration: CRD42024628157), we systematically searched PubMed and APA PsycINFO for studies published between 2014 and 2024. Inclusion criteria encompassed human studies using neuroimaging and ML methods applied to AN, BN, or BED. Data extraction focused on study design, imaging modalities, ML techniques, and performance metrics. Quality was assessed using the GRADE framework and the ROBINS-I tool.

**Results:**

Out of 185 records screened, 5 studies met the inclusion criteria. Most applied support vector machines (SVMs) or other supervised ML models to structural MRI or diffusion tensor imaging data. Cortical thickness alterations in AN and diffusion-based metrics effectively distinguished ED subtypes. However, all studies were observational, heterogeneous, and at moderate to serious risk of bias. Sample sizes were small, and external validation was lacking.

**Conclusion:**

ML applied to neuroimaging shows potential for improving ED characterization and outcome prediction. Nevertheless, methodological limitations restrict generalizability. Future research should focus on larger, multicenter, and multimodal studies to enhance clinical applicability.

*Level of Evidence*: Level IV, multiple observational studies with methodological heterogeneity and moderate to serious risk of bias.

## Introduction

Eating disorders (EDs) are serious psychiatric illnesses associated with high morbidity and mortality and characterized by overlapping clinical features such as distorted body image, obsessive–compulsive behaviors, and impaired insight, which contribute to diagnostic challenges and potentially inappropriate treatment [1]. These shared features suggest the involvement of common underlying mechanisms, including psychological traits such as perfectionism and impulsivity, and neurobiological alterations in fronto-striatal circuits involved in reward processing, inhibitory control, and body image perception.

While this categorical framework facilitates clinical diagnosis and treatment planning, it may oversimplify the substantial heterogeneity observed within and across EDs.

In clinical practice, individuals often exhibit features that span multiple diagnostic categories or evolve over time from one form of ED to another. This phenotypic overlap suggests the existence of shared transdiagnostic dimensions or biotypes that cut across DSM-defined boundaries. Moreover, traits such as compulsivity, perfectionism, and emotional dysregulation are common to many ED presentations, yet their neurobiological underpinnings may vary. This raises two important questions: Do similar clinical features imply shared biological mechanisms? Or might similar behaviors arise from distinct pathophysiological pathways?

These issues challenge the assumption that DSM-defined categories reflect discrete biological entities, and support a shift toward data-driven, dimensional models that incorporate neurobiological, cognitive, and affective dimensions. Recent advances in neuroimaging and machine learning offer promising avenues to identify latent biotypes and to disentangle the biological heterogeneity underlying ED symptomatology. Addressing these open questions is essential for improving diagnostic precision and developing targeted, personalized interventions.

Anorexia nervosa (AN) is a chronic psychiatric disorder characterized by decreased caloric intake, weight loss, distorted body image, and excessive physical activity. Approximately 90% of individuals with this disorder are females [2]. AN carries a high mortality risk due to starvation-related complications [3].

Bulimia nervosa (BN) is an eating disorder that typically begins in adolescence, up to 3% of females and more than 1% of males suffer from this disorder during their lifetime. It is associated with an increased risk for a range of future physical and mental health problems. It is characterized by recurrent binge eating episodes, where individuals consume large amounts of food within a short time and feel a lack of control. They may engage in harmful compensatory behaviors to avoid weight gain, such as self-induced vomiting, laxative misuse, fasting, or excessive exercise. These behaviors occur at least once a week for 3 months, with self-evaluation heavily influenced by body shape and weight [4].

Binge eating disorder (BED) is characterized by recurrent episodes of compulsive overeating (significantly larger amounts of food than most would eat in a short time) accompanied by a feeling of loss of control [5].

The diagnosis of EDs, including AN, BN, and BED, currently relies on a multifaceted approach integrating medical evaluations, psychological assessments, and the application of standardized diagnostic criteria [6]. This process involves a comprehensive assessment using physical examinations, laboratory tests, structured clinical interviews, validated questionnaires, and self-reported measures to capture the complexity of ED symptomatology [7]. While other feeding and eating disorders, such as avoidant/restrictive food intake disorder (ARFID), pica, and rumination disorder, are recognized in the DSM-5, the present review focuses on AN, BN, and BED due to their more consistent representation in the current neuroimaging and machine learning literature. However, the etiology of EDs remains incompletely understood, underscoring the need for more precise diagnostic tools and improved treatment strategies [8]. The development of EDs arises from a complex interplay of genetic, biological, psychological, and socio-environmental factors [9]. Genetic factors significantly influence the heritability of EDs, impacting gender distribution, metabolic traits, the co-occurrence of EDs with other psychiatric conditions, and the neurobiological mechanisms regulating appetite and body weight [10–12]. A wide array of psychological factors contributes, including personality traits such as perfectionism, low self-esteem, impulsivity, and compulsiveness, as well as comorbid psychiatric disorders, such as depression, anxiety disorders, and borderline personality disorder [13–15]. Socio-environmental influences also play a critical role, encompassing aspects of family dynamics, childhood weight status, trauma, peer pressure, and broader societal factors [16–18]. These factors vary across ED subtypes: for example, higher educational attainment is more strongly associated with AN, food insecurity with binge eating disorder, and exposure to social media or video gaming with BED; participation in elite sports (i.e., long-distance running, gymnastics, and high board diving) may also be a contributing factor [9, 18, 19]. A particularly challenging aspect of research in EDs lies in understanding how these diverse psychosocial factors interact with biological mechanisms to shape the neurobiology of these conditions. Moreover, it remains critical to discern the extent to which neurobiological traits are specific to EDs diagnosis. Addressing these questions is essential not only to deepen our understanding of the underlying neurobiological mechanisms of EDs, but also to inform the development of more targeted therapeutic approaches.

Studies employing these modalities consistently report brain alterations across multiple scales, encompassing global brain organization alongside specific disruptions in neural networks involved in key functions, such as reward processing, habit formation, and body perception [20, 21]. Patients with AN have shown hyperactivation of the left parietal and right superior frontal areas in response to a neutral or positive stimulus, whereas patients with BN have shown hyperactivation of the right temporal and right occipital areas Alterations in the cortico-striatal circuits of individuals with BN and BED are similar to those reported in studies of people with substance abuse, with changes in the function of the prefrontal, insular cortex, orbitofrontal cortex (OFC) and striatum. Alterations in the cortico-striatal circuits of individuals with BN and BED are similar to those reported in the studies of people with substance abuse, with changes in the function of the prefrontal, insular cortex, orbitofrontal cortex (OFC), and striatum [18]. In BED, functional imaging studies suggest a shift from ventral-striatal reward-based responses to dorsal-striatal engagement associated with impulsive and compulsive food consumption [22]. However, this neurobiological model remains preliminary and should be interpreted with caution due to limited and heterogeneous evidence. In BN, functional neuroimaging studies suggest that the urge to binge eat may be mediated by hyperactivity of the orbitofrontal cortex (OFC) and anterior cingulate cortex (ACC), along with impaired inhibitory control from lateral prefrontal circuits. Some studies also report hyperactivity in parieto-occipital regions and reduced activation of executive control networks compared to healthy controls [22, 23]. However, these findings should be interpreted with caution due to the moderate to serious risk of bias, small sample sizes, and heterogeneity in imaging protocols and analysis methods [22, 23].

The neurobiology of EDs has been extensively investigated using a range of neuroimaging techniques, including structural imaging to study both gray and white matter, and functional imaging methods [24, 25]. Studies employing these modalities consistently report brain alterations across multiple scales, encompassing global brain organization alongside specific disruptions in neural networks involved in key functions, such as reward processing, habit formation, and body perception [20, 21]. Resting-state functional connectivity (RSFC) analysis provides a robust neuroimaging approach that is less dependent on task compliance, offering a powerful tool for differentiating state-related (e.g., due to malnutrition) from trait-related neural effects [26, 27]. However, despite the significant insights these methods provide, identifying specific neurobiological biomarkers to aid in diagnosis remains a critical area of ongoing research. This challenge is also underscored by recent evidence from Bracké et al. [28], who reviewed longitudinal neuroimaging studies in AN and emphasized the potential role of specific brain-based markers in predicting clinical trajectories and treatment outcomes [28].

Thus, current diagnostic practices heavily rely on clinical observation, behavioral assessments, and self-reported symptoms, underscoring the urgent need for more objective and quantifiable diagnostic tools that complement existing approaches and enhance diagnostic precision. Artificial intelligence (AI) refers to the broader scientific field concerned with building systems that can perform tasks typically requiring human intelligence, such as reasoning, problem-solving, and learning. Machine learning (ML), a subfield of AI, focuses specifically on algorithms that learn patterns from data and improve their performance over time without being explicitly programmed. While all ML is part of AI, not all AI relies on ML; symbolic reasoning and rule-based systems represent other approaches within AI. The advent of sophisticated statistical learning (SL) methods, coupled with readily available computational resources and the emergence of large datasets, has enabled the application of machine learning (ML) to a broad range of healthcare challenges. In this manuscript, we use the term “machine learning” as a broad category that includes both traditional approaches (e.g., regularized regression, decision trees) and more advanced, non-linear models (e.g., support vector machines, neural networks). “Deep learning” (DL), in contrast, is a subfield of ML based on artificial neural networks with multiple hidden layers, capable of learning complex hierarchical representations directly from raw data. While the boundaries between SL, ML, and DL are not always sharply defined, we adopt this taxonomy to enhance clarity and consistency throughout the review. ML, particularly deep learning (DL), holds considerable promise for enhancing ED diagnosis and treatment through its ability to identify complex interactions among numerous risk factors, predict ED development, and personalized treatment strategies. While some ML algorithms particularly deep neural networks and ensemble methods are often considered “black boxes”, many others, including penalized regressions and decision trees, offer greater transparency. Furthermore, recent developments in model-agnostic interpretability methods, such as SHAP and LIME, allow for meaningful assessment of variable importance, thus enhancing the clinical applicability and trustworthiness of even complex ML models. However, the inherent complexity of ML models can potentially limit transparency and reduce confidence among clinicians [29, 30]. Therefore, the focus should be on developing and validating explainable ML models that can improve accuracy while maintaining transparency and clinical utility. This systematic review aims to synthesize and critically evaluate recent studies that apply machine learning techniques to neuroimaging data for the diagnosis, characterization, and outcome prediction of eating disorders. Specifically, it examines the feasibility, accuracy, and clinical relevance of ML models in identifying neurobiological markers associated with EDs.

## Methods

The present review was conducted following the Preferred Reporting Items for Systematic Reviews and Meta-Analyses (PRISMA) statement (registration no CRD42024628157).

### Search strategy and study eligibility criteria

A systematic literature search was conducted using PubMed and APA PsycINFO, which were selected for their high specificity in indexing peer-reviewed studies in medicine, neuroscience, and psychology. Although broader databases such as Scopus were considered, they were not included due to substantial overlap in retrieved records and a lower specificity for the clinical and neuroimaging domains relevant to this review. Articles published between January 1, 2014, and November 30, 2024, were included. This time frame was chosen to capture studies that reflect the application of contemporary machine learning techniques and modern neuroimaging protocols, ensuring methodological consistency and clinical relevance across included papers. We used the following search terms: (“Anorexia Nervosa” OR “Bulimia Nervosa” OR “Binge Eating Disorder” OR “Eating Disorder”) AND (“Neuroimaging” OR “Magnetic Resonance Imaging” OR “Diffusion Tensor Images” OR “Resting State”) AND (“Artificial Intelligence” OR “Machine Learning”). We opted to use the full forms of technical terms (e.g., “Magnetic Resonance Imaging” instead of “MRI”, and “Machine Learning” instead of “ML”) to maximize compatibility with controlled vocabularies such as MeSH and Thesaurus terms in PubMed and PsycINFO. This approach was intended to enhance retrieval precision and reduce ambiguity, while still capturing all relevant literature within the scope of the review.

**Fig. 1:**
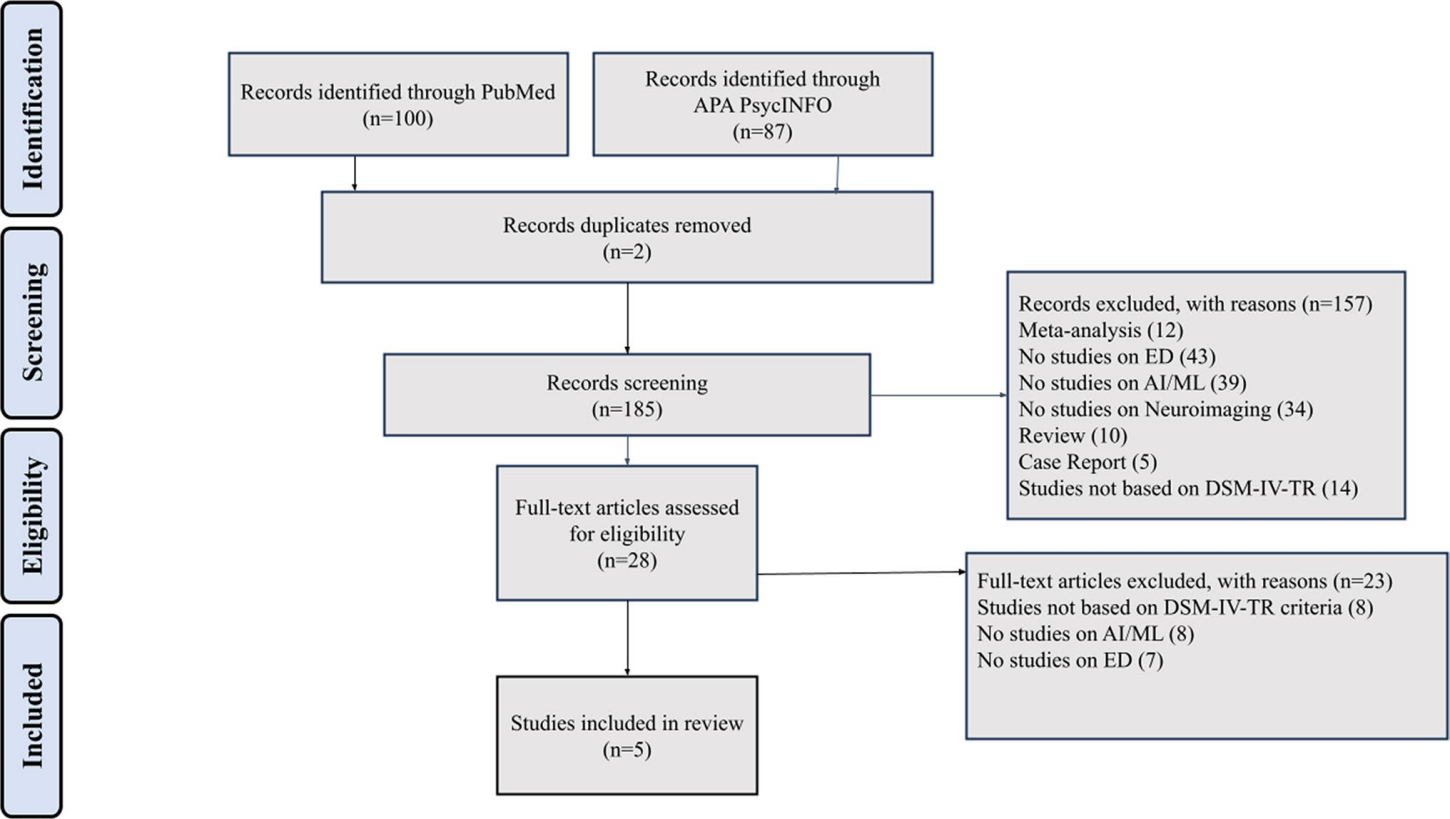
PRISMA search process

Studies were chosen based on these inclusion criteria: randomized controlled trial, retrospective study, cross-sectional study, cohort study, open study, expert opinion, concerning conceptualization, diagnosis of anorexia nervosa, bulimia nervosa, binge eating disorder according to DSM-5-TR, neuroimaging, magnetic resonance imaging, diffusion tensor image, resting-state functional MRI, artificial intelligence and machine learning techniques, studies published in English, studies carried out in humans and studies published in journals indexed in Embase or Medline. The exclusion criteria were meta-analysis, review, duplicates, comments, study protocol, editorials, case reports/case series, theses, proceedings, letters, short surveys and notes, studies irrelevant to the topic, unavailable full-text, and studies that do not meet inclusion criteria. The study selection process was conducted systematically to ensure rigor and transparency, as illustrated in the PRISMA flow diagram (Fig. 1). A comprehensive search was performed in two large databases: PubMed (*n* = 100) and APA PsycINFO (*n* = 87). After removing duplicates (*n* = 2), 185 records remained for screening. During the screening phase, titles and abstracts were assessed for relevance based on predefined inclusion criteria. A total of 157 records were excluded at this stage for the following reasons: 12 were meta-analyses, 10 were reviews, 5 were case reports, 14 were studies not based on DSM-5-TR criteria, 43 were no studies on ED, 39 did not include studies on AI/ML, 34 did not include studies on neuroimaging. Subsequently, 28 full-text articles were assessed for eligibility. Of these, 23 articles were excluded, 8 were studies not based on DSM-5-TR criteria, 8 were not a study on AI/ML, 7 were not studies on ED. Ultimately, 5 studies met all inclusion criteria and were included in the systematic review.

A detailed summary of the included studies, including study design, participant characteristics, and intervention details, is presented in Table 1 for further reference.

**Table 1:** Summary of the included studies

Author	Year and country	Period	Study design	Study sample	Measures	ML model	Diagnosis	Outcome	GRADE assessment of articles
Arold et al	2023, Germany	From 2.78 months between baseline and post-treatment scans to a maximum of 53.54 months post-recovery, with a minimum of 12 months of stable weight recovery	Observational study using structural MRI data. Support vector machine (SVM) machine learning models were applied to cortical thickness and subcortical volume data to classify individuals with anorexia nervosa at different recovery stages from healthy controls and to predict 1-year treatment outcomes	*N* = 573 females: 271 with anorexia nervosa (165 acAN-TP1, 115 acAN-TP2, 89 recAN) and 302 healthy controls (HC)	EDI-2; BDI-2	Support vector machine (SVM) with L2-regularization applied to cortical thickness and subcortical volume features, using PCA for dimensionality reduction and nested cross-validation for performance optimization	Anorexia nervosa (AN) diagnosed according to DSM-5 criteria	Significant classification of underweight and partially weight-restored AN patients from healthy controls; machine learning-based risk score at partial weight restoration predicted 1-year outcome independent of BMI; no significant brain alterations in long-term recovered patients; persistent brain alterations associated with worse outcomes	Low
Cerasa et al	2015, Italy	From 16 months (mean illness duration) to approximately 24 months, with data collected between 2011 and 2012	Observational cross-sectional study using structural MRI. SVM with PCA applied to whole-brain images to classify ED from HC based on neuroanatomical features	*N* = 34 females: 17 with eating disorders (6 anorexia nervosa, 11 bulimia nervosa) and 17 BMI-matched healthy controls	EDI-2; TEC; DES-II; SDQ-20; PBI; EAT-26; BIDA; HAM-A; BDI	Support vector machine (SVM) with linear kernel applied to structural MRI data. PCA used for feature extraction and dimensionality reduction. Model evaluated with 20-fold and tenfold cross-validation	Anorexia nervosa (AN) and bulimia nervosa (BN) diagnosed according to DSM-5 criteria	SVM achieved ≥ 0.80 accuracy distinguishing ED from healthy controls. Discriminative brain regions included occipital cortex, posterior cerebellar lobule, precuneus, sensorimotor/premotor cortices, and medial prefrontal cortex. No global brain volume differences detected	Low
Lavagnino et al	2015, Italy & USA	Cross-sectional study with data collected from AN outpatients and controls in Turin, Italy	Observational study using structural MRI. LASSO machine learning model applied to neuroanatomical volumes to classify AN patients vs. healthy controls and explore associations with symptoms	*N* = 30 females: 15 with anorexia nervosa (12 restricting type, 3 binge-purging) and 15 matched healthy controls	EDI-2; SCID	Least Absolute Shrinkage and Selection Operator (LASSO), with leave-one-out cross-validation, using volumetric features extracted from T1-weighted MRI scans	Anorexia nervosa (DSM-IV-TR)	83.3% classification accuracy (sensitivity 86.7%, specificity 80.0%). Prediction probability correlated with drive for thinness (*r* = 0.52) and BMI (*r* = −0.45). Brain regions contributing to classification included cerebellum, choroid plexus, putamen, accumbens, diencephalon, and third ventricle. Findings suggest neuroimaging may help in clinical prediction	Moderate
Lavagnino et al	2018, USA	Cross-sectional; scans performed after 1–2 weeks of inpatient or partial hospitalization treatment in AN patients	Observational study using structural MRI; machine learning model applied to cortical thickness data to classify participants	*N* = 67 women: 19 with AN (restricting type), 24 recovered from AN (REC-AN), 24 healthy controls (HC)	EDI-3; STAI	Relevance Vector Machine (RVM), linear kernel; applied to cortical thickness from 24 regions (orbitofrontal, insula, frontal, cingulate) using LOOCV	Anorexia nervosa (DSM-IV)	74% sensitivity and 74% specificity in classifying AN vs. HC; cortical thickness patterns (higher in orbitofrontal and insular, lower in superior frontal regions) may serve as state biomarkers; REC-AN group not distinguishable from HC	Moderate
Zhang et al	2024, China	From approximately 30–35 months of illness duration, with no treatment in the previous 12 months	Observational cross-sectional study using diffusion tensor imaging (DTI). Linear SVM applied to diffusion metrics to classify anorexia nervosa and bulimia nervosa patients with leave-one-out cross-validation	*N* = 58 drug-naïve females: 24 with anorexia nervosa and 34 with bulimia nervosa	SCID	Support vector machine (SVM) with linear kernel applied to DTI metrics (AD, FA, MD, RD), evaluated using leave-one-out cross-validation and ROC analysis	Anorexia nervosa (AN) and bulimia nervosa (BN) diagnosed according to DSM-5 criteria	The AD-based SVM model distinguished AN from BN with 75.86% accuracy (sensitivity: 66.67%, specificity: 88.23%, AUC: 0.793). AN patients showed lower AD values in the left middle temporal gyrus (MTG_L) and left superior temporal gyrus (STG_L). No significant differences were found with FA, MD, or RD	Low

*SVM* support vector machine, *HC* healthy controls, *PCA* principal component analysis, *EDs* eating disorders, *AN* anorexia nervosa, *BN* bulimia nervosa, *BDD* body dysmorphic disorder, *BMI* body mass index, *MRI* magnetic resonance imaging, *fMRI* functional magnetic resonance imaging, *DTI* diffusion tensor imaging, *AD* axial diffusivity, *FA* fractional anisotropy, *MD* mean diffusivity, *RD* radial diffusivity, *ROC* receiver operating characteristic, *MTG-L* left middle temporal gyrus, *STG-L* left superior temporal gyrus, *acAN-TP1* acute anorexia nervosa—time point 1, *recAN* recovered anorexia nervosa, *TEC* Traumatic Experiences Checklist, *DES-II* Dissociative Experiences Scale—Version II, *SDQ-20* Somatoform Dissociation Questionnaire—20 items, *PBI* Parental Bonding Instrument, *EAT-26* Eating Attitude Test—26 items, *BIDA* Body Image Dimensional Assessment, *EDI-2* Eating Disorder Inventory—Version 2, *EDI-3* Eating Disorder Inventory—Version 3, *HAM-A* Hamilton Anxiety Rating Scale, *BDI* Beck Depression Inventory, *SCID* Structured Clinical Interview for Diagnosis, *MADRS* Montgomery-Åsberg Depression Rating Scale, *BDD-YBOCS* Yale-Brown Obsessive Compulsive Scale for Body Dysmorphic Disorder, *MINI* Mini International Neuropsychiatric Interview, *BDD Diagnostic Module* Body Dysmorphic Disorder Diagnostic Module, *YBC-EDS* Yale-Brown-Cornell Eating Disorder Scale, *EDE* Eating Disorder Examination, *BABS* Brown Assessment of Beliefs Scale

### Study selection

The selection of studies for this review occurred in a two-stage process. Initially, four independent reviewers assessed the titles and abstracts of all the retrieved papers (CV, EC, AM, SL). In the subsequent stage, these same reviewers individually examined the full texts of the papers identified in the first phase. Any discrepancies between the four reviewers were resolved by involving a senior researcher.

### Data extraction and data synthesis

Four independent researchers (SP, EP, BG, and RM) carried out data extraction for each included study, utilizing a standardized data extraction sheet in Microsoft Excel. The focus of this extraction encompassed several key subjects, including study design, participant characteristics, diagnosis of ED, neuroimaging, and AI and ML techniques details derived from the original research. A meta-analysis was not conducted due to significant heterogeneity in study designs, interventions, outcome measures, and durations. Therefore, a narrative synthesis was employed to summarize the findings systematically.

### Quality assessment

Given the heterogeneity of the included studies, the Grading of Recommendations, Assessment, Development, and Evaluation (GRADE) approach was employed to assess the quality of the evidence. As no randomized controlled trials were selected, the ROBINS-I tool was used. This assessment was conducted by two reviewers (VDS, MDA) with any disagreements resolved through discussion with an additional reviewer (FM) (Table 2).

**Table 2:** This table summarizes the risk of bias assessment for each included study, evaluated using the ROBINS-I tool

Author/year	Confounding bias	Selection bias	Classification bias	Deviations from intended interventions	Missing data bias	Outcome measurement bias	Reporting bias	Overall risk of bias
Arold et al. 2023	Moderate	Moderate	Low	Low	Low	Moderate	Moderate	Moderate
Cerasa et al. 2015	Serious	Serious	Moderate	Low	Low	Moderate	Serious	Serious
Lavagnino et al. 2015	Moderate	Serious	Low	Low	Low	Moderate	Low	Moderate
Lavagnino et al. 2018	Moderate	Serious	Low	Low	Low	Moderate	Low	Moderate
Vaughn et al. 2019	Serious	Serious	Moderate	Low	Low	Serious	Serious	Serious
Zhang et al. 2024	Serious	Serious	Moderate	Low	Low	Moderate	Serious	Serious

Each domain confounding, selection, classification of interventions, deviations from intended interventions, missing data, outcome measurement, and reporting was rated as *Low*, *Moderate*, or *Serious* risk. The overall risk of bias reflects the highest level of concern across domains, following GRADE recommendations

## Results

The application of machine learning (ML) techniques to neuroimaging data in eating disorders (EDs) demonstrates encouraging diagnostic and prognostic potential, particularly in anorexia nervosa (AN). As summarized in Table 1, Cerasa et al. [31] employed support vector machines (SVM) on T1-weighted structural MRI data to classify patients with AN and bulimia nervosa (BN) versus BMI-matched healthy controls (HC), achieving classification accuracies exceeding 80%. The discriminative features involved regions implicated in sensory integration, body image, and interoception, such as the occipital cortex, premotor areas, and medial prefrontal cortex.

Lavagnino et al. [32] applied the Least Absolute Shrinkage and Selection Operator (LASSO) to volumetric features extracted via FreeSurfer, identifying six neuroanatomical structures—including the putamen, cerebellum, and accumbens that significantly predicted AN diagnosis. The probabilistic outputs of the model correlated with key clinical features (e.g., BMI and drive for thinness), suggesting biological validity. In a subsequent study, Lavagnino et al. [33] examined cortical thickness across 24 regions and used a relevance vector machine (RVM) classifier to differentiate AN, recovered AN (REC-AN), and HC, yielding moderate predictive performance (74% sensitivity and specificity). Findings pointed to cortical asymmetries, particularly in orbitofrontal and insular areas, as potential state-dependent biomarkers of AN.

Arold et al. [34] investigated whether brain structural alterations observed after partial weight restoration in AN could serve as predictors of long-term outcomes. ML models successfully distinguished acutely underweight and partially weight-restored patients from HC, but failed to classify fully recovered individuals, suggesting partial normalization of neuroanatomical alterations. Persistent changes, particularly in regions of high functional connectivity (e.g., insula, orbitofrontal cortex), were associated with poorer clinical trajectories, indicating potential prognostic value.

Zheng et al. [35] was the only study utilizing diffusion tensor imaging (DTI). Their SVM model, trained on axial diffusivity (AD) features, achieved 75.9% accuracy in distinguishing AN from BN, with key contributions from the left middle and superior temporal gyri. No significant group differences emerged in fractional anisotropy or mean diffusivity, suggesting AD as a more sensitive metric in this context.

According to studies, predictive accuracies ranged from 74 to 83.3% (Table 1), with most models relying exclusively on structural MRI and internally validated through cross-validation techniques. As detailed in Table 3, there was minimal integration of psychometric, genetic, or environmental data, which limits both model generalizability and translational applicability. These findings underscore the potential of ML-based neuroimaging for ED characterization while highlighting the need for more integrative, multimodal approaches to capture the full biopsychosocial complexity of these disorders.

**Table 3:** Overview of included studies by neuroimaging modality, machine learning approach, and data integration strategy

Study	Neuroimaging modality	ML technique	Psychometric measures included	Genetic/environmental data	Multimodal integration
Arold et al. [34]	Structural MRI (cortical thickness, subcortical volumes)	SVM with PCA and L2-regularization	EDI-2, BDI-2	No	Partial (structural MRI + clinical outcome prediction)
Cerasa et al. [31]	Structural MRI (whole brain)	SVM with PCA (linear kernel)	EDI-2, TEC, DES-II, SDQ-20, PBI, EAT-26, BIDA, HAM-A, BDI	Environmental measures (e.g., parental bonding, trauma)	Yes (neuroimaging + extensive psychometric profiling)
Lavagnino et al. [32]	Structural MRI (volumetric T1-weighted)	LASSO regression	EDI-2, SCID	No	Limited (neuroimaging + symptoms)
Lavagnino et al. [33]	Structural MRI (cortical thickness)	Relevance vector machine (RVM)	EDI-3, STAI	No	Limited (neuroimaging + symptom severity)
Zhang et al. [36]	Diffusion tensor imaging (DTI)	SVM (linear kernel)	SCID	No	Minimal (neuroimaging only)

## Discussion

EDs are severe psychiatric illnesses associated with substantial morbidity and mortality risk. Current diagnostic practices, reliant on clinical observation, structured interviews, standardized questionnaires, and self-reported measures, struggle to fully capture the complicated and polyhedral nature of EDs, leading to diagnostic challenges and potentially suboptimal treatment strategies [7]. The etiology of EDs is complex, arising from a dynamic interplay of genetic predisposition, neurobiological vulnerabilities, psychological factors, and socio-environmental influences, the relative contributions of which vary significantly across ED subtypes [37]. Genetic factors influence heritability, gender distribution, metabolic traits, comorbidity with other psychiatric disorders, and the neurobiological regulation of appetite and body weight. Psychological factors include personality traits (perfectionism, low self-esteem, impulsivity, compulsivity), and the presence of comorbid conditions such as depression, anxiety, and borderline personality disorder [38]. Crucially, socio-environmental factors, including family dynamics, childhood weight status, trauma, peer pressure, and broader societal influences, exert a profound impact, particularly concerning exposure to media and social pressures related to body image [17]. The distinction interplay of these factors across ED subtypes further complicates diagnostic clarity: for instance, higher educational attainment is more strongly associated with AN, food insecurity with BED, and social media exposure with BED [39]. Understanding how these diverse factors interact with underlying biological mechanisms to shape the neurobiology of EDs remains a critical, yet unresolved, challenge. Furthermore, identifying neurobiological traits specific to ED diagnoses is predominant for developing effective, targeted treatments. Neuroimaging studies, employing various techniques from structural and functional MRI [31–33] to DTI [35], have consistently revealed brain alterations across multiple scales, from global brain organization to highly specific disruptions within neural networks involved in reward processing, habit formation, and body perception. While functional neuroimaging provides valuable insights into brain activity patterns, RSFC analysis offers a powerful, less task-dependent approach to disentangling state-related (e.g., effects of malnutrition) from trait-related neural effects. Despite these advances, translating these neuroimaging findings into reliable, clinically useful biomarkers remains a key challenge. However, while these findings highlight promising neurobiological associations, caution is warranted in interpreting them as direct reflections of clinical phenotypes. Eating disorders emerge from the complex interaction of biological, psychological, and socio-environmental factors, which vary across individuals and over time. The risk of oversimplifying such complexity by attributing symptomatology solely to brain alterations must be acknowledged. Neural markers should be understood not as definitive diagnostic entities but as one layer within a multidimensional framework of psychopathology [40, 41]. Future studies should strive to integrate neuroimaging data with ecological, developmental, and experiential factors better to capture the fluid and heterogeneous nature of EDs [42, 43]. This integrative approach is essential to avoid the pitfalls of reductionism and to ensure that neurobiological insights meaningfully inform person-centered care. Current diagnostic practices rely heavily on clinical observation and self-reported symptoms, underscoring the urgent need for objective, quantifiable diagnostic tools to complement existing approaches and improve diagnostic precision.

The integration of sophisticated statistical learning (SL) and ML methods [44], particularly DL techniques, offers considerable promise for revolutionizing ED diagnosis and treatment. ML's capacity to identify complex, non-linear interactions among numerous risk factors, predict ED development, and personalize treatment strategies, is particularly relevant given the multifaceted nature of EDs. However, the inherent "black box" nature of complex ML models can limit transparency and erode clinician confidence. Therefore, a concerted effort should focus on developing and rigorously validating *explainable* ML models that enhance diagnostic accuracy while maintaining transparency and clinical utility.

This review presents several limitations that warrant careful consideration. Moreover, the overall body of evidence is characterized by a high risk of bias and substantial uncertainty, due to small sample sizes, lack of pre-registered protocols, and variability in study quality. These factors warrant caution in the interpretation of reported results and highlight the need for more rigorous study designs and external validation in future research. Firstly, the included studies exhibited significant heterogeneity in terms of sample size, participant demographics (age, gender, ethnicity, comorbidity), and methodologies (neuroimaging modalities, machine learning algorithms, data preprocessing techniques, and outcome measures). This heterogeneity makes a direct comparison of findings challenging and limits the ability to draw robust, universally applicable conclusions. The selection of studies was also influenced by the availability of published data, potentially introducing bias. Secondly, the application of ML models, while promising, introduces inherent complexities and potential limitations that need scrutiny. The "black box" nature of some ML algorithms, particularly DL models, can hamper transparency and reduce clinician confidence in their use for diagnostic and prognostic purposes. Furthermore, the potential for bias in ML models arising from data imbalances, confounding factors, and limitations in model generalizability requires thorough attention. Moreover, future studies should explicitly address the clinical implications of misclassification errors in ML applications. In particular, the potential harm of false positives such as incorrectly labeling a vulnerable individual as at risk for AN may contribute to unnecessary stigma, psychological distress, or inappropriate interventions [45–47]. A transparent discussion of model uncertainty and classification thresholds is essential for safe and responsible clinical deployment. Bias arising from non-representative samples, poorly chosen features, and inadequate model validation could lead to inaccurate and potentially misleading clinical predictions. Furthermore, the limited number of studies included in this review reflects the emerging and highly specialized nature of research combining neuroimaging and machine learning in the context of eating disorders. Despite growing interest in each individually, few studies have integrated these methodologies with sufficient methodological rigor to meet inclusion criteria. This gap underscores the need for future research adopting robust, interdisciplinary designs to advance the field. In addition, the absence of standardized neuroimaging preprocessing and analysis pipelines represents a major limitation for cross-study comparability and replication. Variability in motion correction, spatial normalization, or segmentation procedures may substantially affect extracted features and model performance. To enhance reproducibility, future studies should adopt transparent, open-source pipelines, report preprocessing steps in detail, and validate models across independent datasets processed with different pipelines. Achieving robust and generalizable findings in this field will likely require large-scale, multicenter collaborations. Such efforts enable access to larger and more diverse samples, increase statistical power, and facilitate external validation across heterogeneous populations and imaging protocols. However, designing and implementing multicenter studies poses several challenges, including data harmonization, ethical and legal constraints in data sharing, variability in imaging acquisition parameters, and the need for consensus on standardized preprocessing pipelines. Future initiatives should prioritize open science frameworks, federated learning approaches, and collaborative consortia to overcome these obstacles and accelerate progress in ML-based neuroimaging research on EDs.

Thirdly, translating the promising research findings presented into clinically relevant tools and effective interventions demands further robust investigation and rigorous validation. Large-scale, well-designed, multi-center studies employing standardized diagnostic criteria, outcome measures, and data acquisition protocols are critical for establishing the clinical utility and generalizability of the neuroimaging and ML approaches reviewed. The development of user-friendly interfaces and clinically interpretable algorithms is also necessary to facilitate widespread adoption and integration into routine clinical practice. Finally, the integration of other relevant data, such as genetic information, psychological assessments, and environmental factors, could potentially improve the accuracy and precision of ED diagnosis and risk prediction models.

## Strengths and limits

This systematic review has several strengths. It is the first to synthesize and critically evaluate the integration of neuroimaging and machine learning in eating disorders, following PRISMA guidelines and including only peer-reviewed human studies indexed in major biomedical databases. The review highlights key methodological trends and limitations, offering clinically relevant insights for future research. However, the study has some limitations. The number of eligible studies was limited, and all were observational, with moderate to serious risk of bias. The included studies showed high heterogeneity in sample size, imaging modalities, and machine learning approaches, precluding meta-analytic synthesis. Moreover, the generalizability of the findings is constrained by small sample sizes and a lack of external validation.

## What is already known on this subject?

Previous studies have shown that individuals with eating disorders exhibit consistent neurobiological alterations, particularly in fronto-striatal and limbic circuits. While neuroimaging and machine learning have been increasingly applied to psychiatric disorders, their combined use in the study of eating disorders remains limited, methodologically heterogeneous, and lacking in clinical translation.

## What your study adds

This study provides the first systematic synthesis of research applying machine learning techniques to neuroimaging data in eating disorders. It identifies promising diagnostic and prognostic applications while highlighting critical methodological limitations, such as small sample sizes, lack of external validation, and high risk of bias. These findings underscore the need for future large-scale, multimodal studies to improve translational applicability in clinical and public health settings.

## Conclusion

EDs represent a significant public health concern, demanding improved diagnostic tools and treatment strategies. While traditional diagnostic methods rely heavily on clinical observation and self-reported symptoms, neuroimaging techniques offer the potential for more objective and precise biomarkers. This review highlights the growing application of ML to neuroimaging data in ED research, demonstrating its potential to enhance diagnostic accuracy and personalized treatment. Studies utilizing MRI have shown success in distinguishing AN from healthy controls, identifying brain regions involved in AN pathophysiology, and even predicting long-term outcomes. The investigation of cortical thickness patterns revealed unique signatures characteristic of AN. Furthermore, the use of DTI demonstrated promise in differentiating AN from BN. However, considerable challenges remain. The heterogeneity of existing studies, including variations in sample size, participant characteristics, and methodological approaches, hampers the ability to draw universally applicable conclusions. The inherent complexity of ML models necessitates a focus on explainable AI to ensure transparency and build clinician confidence. Furthermore, rigorous validation in large, well-powered, multi-site studies is crucial to confirm these findings and ensure the generalizability of ML-based diagnostic tools.

## Future direction

Future directions in EDs research should cover several main areas dedicated to intensifying our understanding, refining diagnosis, and improving treatment outcomes for these complex conditions. One of the principal priorities should be the establishment of longitudinal studies that meticulously track changes in neural, psychological, and behavioral patterns over time. Such research attempts will not only illuminate causal relationships, but also help to identify critical intervention periods, particularly within vulnerable populations such as adolescents, who may be at heightened risk for developing EDs during pivotal developmental stages. Machine learning approaches may also be applied to longitudinal neurocognitive data to characterize trajectories of cognitive recovery following nutritional rehabilitation, potentially identifying predictive markers of treatment response [48, 49].

Future studies should also emphasize external validation of ML models across independent, diverse clinical samples to ensure generalizability and replicability of findings, addressing current concerns about overfitting and model robustness [50].

Additionally, the development of personalized treatment strategies is crucial, leveraging predictive modeling and ML techniques to enhance intervention effectiveness. By tailoring therapeutic approaches to meet the specific needs of individuals, researchers can better target the unique characteristics of each patient's condition, potentially leading to more successful outcomes. Future research should also prioritize the integration of psychological, behavioral, and socio-environmental variables into ML models, to avoid overemphasizing the biological substrate of EDs and to better reflect their inherently multidimensional nature [41, 43]. The future integration of predictive ML models into digital platforms could facilitate more personalized and scalable interventions for EDs. Although not directly addressed in the current review, the deployment of such tools within telehealth environments may eventually support early detection and clinical monitoring, especially in underserved populations. These translational potential underscores the importance of interdisciplinary collaboration between computational scientists, clinicians, and digital health developers. This multidimensionality includes potential interactions between biological vulnerabilities and environmental stressors, which are increasingly recognized as key to understanding ED onset and progression. Many ML algorithms, particularly non-linear models such as random forests, gradient boosting machines, and deep learning architectures, are well-suited to capture high-order interactions without requiring them to be pre-specified. Future studies should explicitly test such interactions by integrating multimodal data sources and applying explainable ML techniques capable of identifying synergistic or moderating effects across biological and psychosocial domains.

As highlighted in reference [41], the integration of environmental, behavioral, and psychological variables into predictive models is essential to fully capture the complexity of eating disorders and to avoid biologically reductionist interpretations. This perspective is aligned with a network-based view of brain function that challenges classical anatomical localism. As Pessoa argues in The Entangled Brain (MIT Press), mental processes emerge from dynamic, distributed neural interactions rather than discrete regional activations, an argument that supports the use of ML models capable of capturing such multivariate brain–behavior relationships.

Furthermore, leveraging technology and telehealth platforms to deliver treatments presents an exciting avenue for significantly expanding access to care. This approach is particularly beneficial for individuals residing in underserved or remote areas, who may currently face barriers to receiving adequate treatment. By facilitating virtual consultations and digital interventions, researchers and clinicians can ensure that more individuals benefit from timely and effective support, ultimately bridging gaps in care. Beyond prediction and prognosis, machine learning approaches may also support the refinement of current diagnostic categories in EDs. By identifying latent biotypes across neurobiological, cognitive, and behavioral domains, ML has the potential to move psychiatric classification toward more dimensional and biologically informed systems that better reflect underlying pathophysiological mechanisms.

Incorporating these directions into future research will not only advance the field of EDs studies, but also result in more comprehensive, effective, and accessible treatment options for those affected by these challenging disorders.

## Data Availability

No datasets were generated or analysed during the current study.

## Appendix

### Glossary of machine learning terms

1. L2 regularization: A technique used to prevent overfitting by penalizing large model coefficients; also known as ridge regression.
2. Linear kernel: A function used in support vector machines (SVM) that assumes a linear relationship between features, allowing for a simple, interpretable decision boundary.
3. Principal component analysis (PCA): A method for reducing the number of input variables in a dataset by transforming them into a smaller set of uncorrelated components that retain most of the variance.
4. Cross-validation: A statistical method for evaluating model performance by dividing data into subsets used for training and testing, helping to estimate generalizability.
5. Leave-one-out cross-validation: A specific form of cross-validation where one observation is used for testing and all remaining observations are used for training; this is repeated for each data point.
6. Performance optimization: The process of adjusting model parameters and structure to maximize accuracy, precision, or other performance metrics based on training data.
